# Methods for quantification of cannabinoids: a narrative review

**DOI:** 10.1186/s42238-020-00040-2

**Published:** 2020-10-09

**Authors:** Masoumeh Pourseyed Lazarjani, Stephanie Torres, Thom Hooker, Chris Fowlie, Owen Young, Ali Seyfoddin

**Affiliations:** 1 Drug Delivery Research Group, School of Science, Faculty of Health and Environmental Sciences, Auckland University of Technology, Auckland, New Zealand; 2 Chapman University, Orange, California USA; 3 ZeaCann LTD, Auckland, New Zealand; 4 School of Science, Faculty of Health and Environmental Sciences, Auckland University of Technology, Auckland, New Zealand

**Keywords:** Cannabis, Cannabinoids, Analytical, THC, CBD, Quantification

## Abstract

**Background:**

Around 144 cannabinoids have been identified in cannabis plant, among them tetrahydrocannabinol (THC) and cannabidiol (CBD) are the most prominent ones. Because of the legal restrictions on cannabis in many countries, it is difficult to obtain standards to use in research; nonetheless, it is important to develop a cannabinoid quantification technique with pharmaceutical applications for quality control of future therapeutic cannabinoids.

**Method:**

To find relevant articles for this narrative review paper, a combination of keywords such as medicinal cannabis, analytical, quantification and cannabinoids were searched for in PubMed, EMBASE, MEDLINE, Google Scholar and Cochrane Library (Wiley) databases.

**Results:**

The most common cannabinoid quantification techniques include gas chromatography (GC) and high-performance liquid chromatography (HPLC). GC is often used in conjunction with mass spectrometry (MS) or flame ionization detection (FID). The major advantage of GC is terpenes quantification however, for evaluating acidic cannabinoids it needs to be derivatised. The main advantage of HPLC is the ability to quantify both acidic and neutral forms of cannabinoids without derivatisation which is often with MS or ultraviolet (UV) detectors.

**Conclusion:**

Based on the information presented in this review, the ideal cannabinoid quantification method is HPLC- MS/MS for the cannabinoids.

## Introduction

*Cannabis sativa* L. is an annual herbaceous flowering plant indigenous to eastern Asia (De Backer et al. 2009). The phenotypes of cannabis are highly variable and the plant is accepted to have two subspecies: *C. sativa* subsp. *sativa* and *C. sativa* subsp. *indica* (Hillig and Mahlberg 2004; Knight et al. 2010). A third subspecies, *C. sativa* subsp. *ruderalis,* has been identified; however, it is not broadly recognized (Fischedick et al. 2010a; Hillig and Mahlberg 2004). Cannabis has been used for its therapeutic properties for thousands of years and it was introduced in western medicine in the nineteenth century until its prohibition in the US from mid-1930s (Aizpurua-Olaizola et al. 2014).

**Fig. 1:**
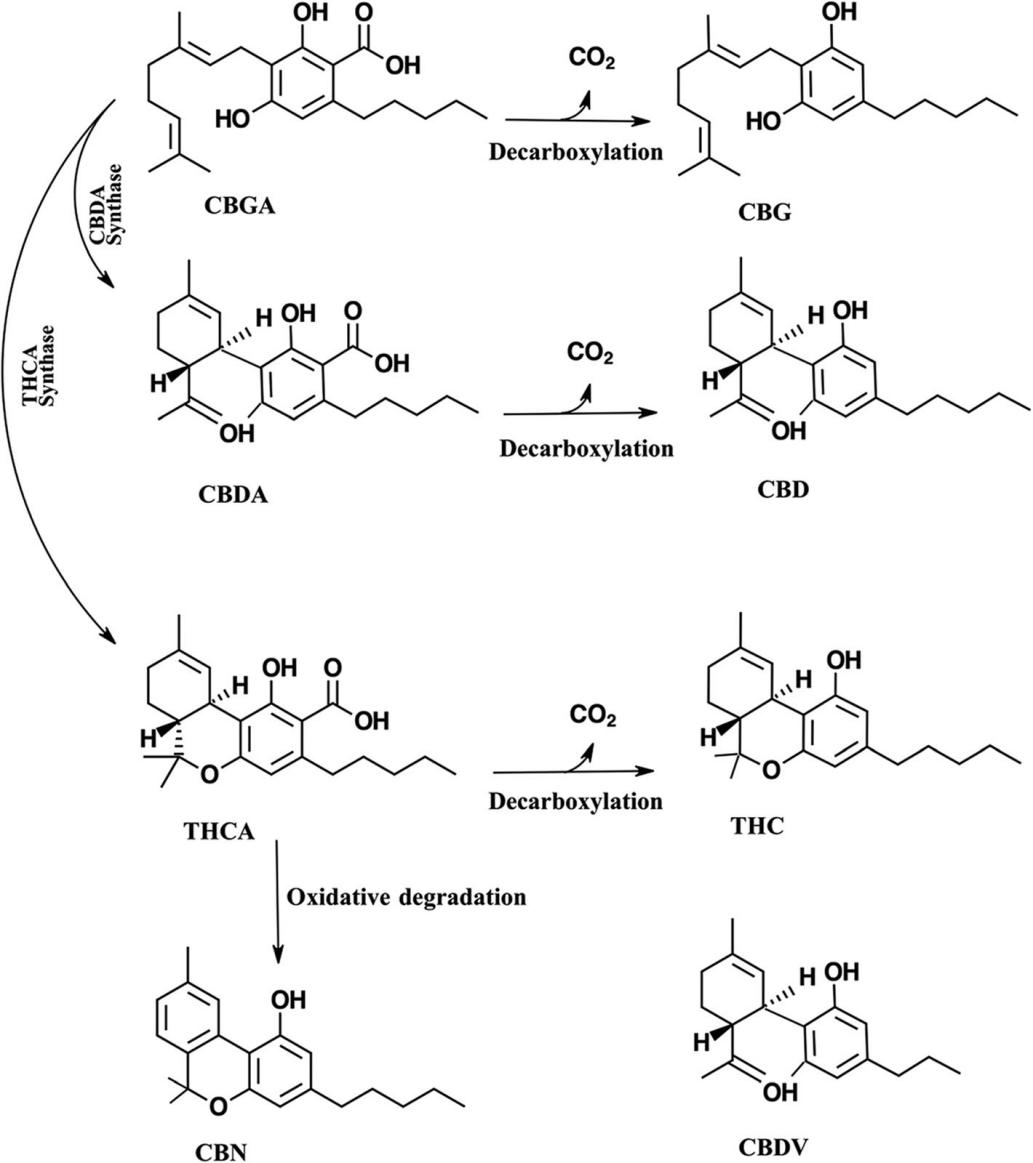
The most common cannabinoids and their conversion pathway by decarboxylation because of heat or aging. C*BGA can convert to CBDA and THCA by CBDA synthase and THCA synthase, respectively. CBGA: cannabigerolic acid, CBG: cannabigerol, CBDA: cannabidiolic acid, CBD: cannabidiol, THCA: tetrahydrocannabinolic acid, THC: tetrahyrocannabinol, CBN: cannabinol (*Fathordoobady et al. 2019*)*

The medicinal compounds from cannabis are mostly concentrated in the female flowers of this dioecious species (Fischedick et al. 2010a). The so-called resin is the source of a wide variety of terpenoids and cannabinoids (Fischedick et al. 2010a). The therapeutic properties of cannabis are attributed to cannabinoids (Hazekamp et al. 2014). Cannabinoids are found in the resin produced by the trichomes which are widely distributed on both the male and female plants however are most highly concentrated on the female flowers of the cannabis plant (Citti et al. 2018; De Backer et al. 2009). Cannabinoids are terpenophenolic compounds unique to cannabis *(*Hillig 2004*)*. To date, 144 cannabinoids have been identified (Hazekamp et al. 2014). The two cannabinoids most well known for their therapeutic properties are tetrahydrocannabinol (THC) and cannabidiol (CBD) (Aizpurua-Olaizola et al. 2016; Hillig 2004). THC and CBD are the neutral homologs of tetrahydrocannabinolic acid (THCA) and cannabidiol acid (CBDA) respectively (Aizpurua-Olaizola et al. 2016). A conventional classification model of cannabinoids is due to their chemical contents dividing them to eleven subclasses including cannabigerol (CBG), tetrahydrocannabinol (THC), cannabidiol (CBD), cannabichromene (CBC), cannabinol (CBN), (−)-Δ8-*trans*-tetrahydrocannabinol (Δ8-THC), cannabicyclol (CBL), cannabinodiol (CBND), cannabielsoin (CBE), cannabitriol (CBT) and miscellaneous (Berman et al. 2018) (Fig. 1).

Because consumers have limited means to analyse the chemical composition of the cannabis products, consumers may be inadvertently purchasing products with undesired properties given that different cannabinoids produce different effects (Fischedick et al. 2010b). As a result, it is important to implement methods of quality control so that consumers can be certain that what they are consuming will have the desired effects (Dussy et al. 2005; Fischedick et al. 2010a; Fischedick et al. 2010b). As cannabis use becomes progressively accepted, it becomes increasingly important to quantify the cannabinoid profile and content of cannabis preparations to ensure the uniformity and quality of the preparations (Omar et al. 2014).

A variety of analytical techniques have been developed for quantification and qualification cannabinoids and other compounds in cannabis plant. Advances in analytical methods have also resulted in detection of various compounds from cannabis extracts in the last decade (eg terpenes). The purpose of this literature review is to explore cannabinoid quantification techniques and subsequently suggest an optimal method for pharmaceutical grade quantification.

## Methods

To find relevant papers for this narrative review paper many data bases have been reviewed for 8 months. A combination of keywords such as medicinal cannabis, analytical, quantification and cannabinoids were searched. Papers from 1967 to 2019 from PubMed, EMBASE, MEDLINE, Google Scholar and Cochrane Library (Wiley) databases have been searched in English. In the next step, papers have been scanned to discard irrelevant papers. Those papers which were relevant went through for more investigations in details. In total, the number of papers which have been read were about 75 including around 15 irrelevant papers.

## Quantitative analysis of cannabinoids

### Gas chromatography (GC)

Gas chromatography (GC) is one of the most commonly used chromatographic methods in quantitative cannabinoid analysis (Hazekamp et al. 2009). Gas chromatography is typically completed in under 20 min at up to 300 °C and makes use of stationary phases with low polarities, such as 5% diphenyl- and 95% dimethyl polysiloxane (Leghissa et al. 2018a). It is important to note that the total quantity of cannabinoids in a sample is the sum of the acidic and neutral components (Citti et al. 2018). Because gas chromatography requires high column temperatures, the acidic cannabinoids undergo decarboxylation during transit through the column (Citti et al. 2018; De Backer et al. 2009; Hazekamp et al. 2009). Thus, acidic cannabinoids cannot be determined unless they are derivatized prior to analysis (Hazekamp et al. 2009). Not only does derivatization preserve cannabinoid structure, but it also causes cannabinoids to become more volatile, thus improving peak shape (Leghissa et al. 2018a). Dussy and Hamberg (2005) suggested calculating the amount of neutral and acidic cannabinoids separately in order to accurately determine the total cannabinoid content. Gas chromatography resolves cannabinoids but detection on elution presents its own challenges and solutions.

#### GC- FID/MS

GC is normally coupled with mass spectrometry (MS) or flame ionization detection (FID) to detect and quantify cannabinoids (Citti et al. 2018; Hazekamp et al. 2009) (Tables 1 and 2). MS employs standardized electron ionization to fragment analytes, permitting the use of compound libraries to identify the parent analyte. FID provides more accurate cannabinoid quantification because it makes use of relatively cheap authentic standards while mass spectrometry usually requires equivalent deuterated standards, which are expensive and not available for all cannabinoids (Citti et al. 2018; Hazekamp et al. 2009).

**Table 1:** An overview to the key properties of common GC-MS method for analysing cannabinoids with a capillary column in six different studies

Key capillary column properties	Cannabinoids analysed	Oven process	Carrier gas	Range	LOD	LOQ	References
Silica capillary column coated with DB1	16 major cannabinoids	Initial 10 °C, 108 °C /min, up to 280 °C Hold for 30 min	Helium	N/A	N/A	N/A	(Hazekamp et al. 2005)
VA5MS capillary column coated with DB1	Δ^9^-THC, CBD, CBN, CBG, THCA, CBGA, CBDA	Initial 100 °C, 108 °C /min, up to 280 °C Hold for 30 min	Helium	N/A	N/A	N/A	(Hazekamp et al. 2009)
5% Cross-linked phenylmethyl siloxane capillary column	CBG, CBD, CBDA, CBN, CBGA, THC, CBC, THCA	Initial 50 °C, 6 °C /min, up to 300 °C, hold for 4 min. (3 min solvent delay was applied)	Helium	m/z^a^ 40–500	N/A	N/A	(Namdar et al. 2018)
5% Diphenyl/95% dimethyl polysiloxane capillary column	THC-THCA	Initial 70 °C, 40 °C /min up to 180 °C, then 10 °C/min up to 300 °C Hold for 6.25 min	Helium	0.10–4.00% (w/w)	0.03% (w/w)	10% (w/w)	(Casiraghi et al. 2018)
Cross-linked poly-5% diphenyl-95% dimethyl polysiloxane capillary column	CBDA-CBD	Initial 45 °C, 2 °C /min, up tp100°C, then 5 °C /min up to 250 °C Hold for 5 min	Helium	*m*/*z* 40–500	N/A	N/A	(Pellati et al. 2018)
5% Cross-linked phenylmethyl siloxane capillary column	CBG, CBD, THC, CBC, CBN	Initial 50 °C, 6 °C/min, up to 300 °C Hold for 4 min, (3 min solvent delay was applied)	Helium	*m*/*z* 40–400	N/A	N/A	(Namdar et al. 2019)

^a^M stand for mass and z is charge number of ions. For GC-MS Z is always almost 1, so M/Z is mass

**Table 2:** Key properties of GC-FID method for analysing cannabinoids with a capillary column in four different studies

Capillary column properties	Cannabinoids analysed	Oven process	Carrier gas	References
DB5 capillary column	THCA, CBGA, CBCA, THC, CBG, CBC	Initial 60 °C, 3 °C/min, up to 240 °C Hold for 5 min	Nitrogen	(Romano and Hazekamp 2013)
Silica capillary column coated with DB1	Δ^9^-THC, CBD, CBN, CBG, THCA, CBGA, CBDA	Initial 100 °C, 108 °C/min, up to 280 °C Hold for 30 min	Nitrogen	(Hazekamp et al. 2009)
DB5 5% diphenyl/95% dimethylpolysiloxane capillary column	THC-THCA	Initial 200 °C, 10 °C/min, up to 300 °C Hold for 2 min	Helium	(Casiraghi et al. 2018)
Cross-linked poly-5% diphenyl-95% dimethyl polysiloxane capillary column	CBDA-CBD	Initial 45 °C, 2 °C/min, up to 100 °C then 5 °C/min, up to 250 °C. Hold for 5 min	Helium	(Pellati et al. 2018)

As it is shown in Table 1, in the all mentioned references the carrier gas is helium in GC-MS because it provides higher efficacy than other gases such as hydrogen and nitrogen. In each study, according to the compound of interest, different column and different protocols were used. The high temperature in injection point is the con for preserving acidic form of cannabinoids. To validate all the quantification methods two parameters should be detected. Limit of detection (LOD) and limit of quantification (LOQ). These two parameters show the lowest concentration of the interest compound that can be reliably measured by an analytical method which are mentioned in Table 1 for GC-MS method.

Leghissa et al. (2018b) used Multiple Reaction Monitoring (MRM) analysis of cannabis from a surrogate hops matrix by GC-MS with triple quadrupole mass spectrometry for the first time. They used silylated cannabinoids to avoid decarboxylation process due to high temperature in GC injection port. They found that this method is applicable to cannabinoids analysis from plant materials and cannabis products. The main achievement of their study is that, in this method, because the risk of interferences from the essential oils and waxes is reduced the extraction need less sample preparation in the laboratories compared to other techniques like SPE.

In another study, Gas Chromatography with Vacuum Ultraviolet spectroscopy (GC-VUV) was used which is gas chromatography with vacuum ultraviolet spectroscopy. The detection of cannabinoids and the cannabinoid metabolites was fast and simple, so that it can be used in rapid detection of them even without having a baseline for cannabinoids for comparison. This method has just one disadvantage which is high limit of detection (LODs). Due to this drawback, detecting analytes in biological matrices cannot be accomplished without pretreatments (Leghissa et al. 2018c).

#### Two-dimensional gas chromatography

Experience has shown that one-dimensional gas chromatography does not provide enough resolution to analyse complex cannabinoid mixtures (Aizpurua-Olaizola et al. 2016). Two-dimensional gas chromatography (GC × GC) has been found to be preferable over one dimensional GC for analyzing complex mixtures, such as cannabis extracts, in that it reveals more sample components (Dallüge et al. 2003; Groger et al. 2008; Omar et al. 2014). Additionally, GC × GC produces two sets of retention data for sample constituents and this can greatly aid analyte identification (Dallüge et al. 2003).

In the Table 2, nitrogen and helium are the carrier gases. In many studies, it is proved that nitrogen has the best efficacy, but it is time consuming. On the other hand, by using helium, the process is rapid and efficient, but the price is not affordable. The Initial and end temperatures, the type of columns and thedrawback are almost similar to GC-MS.

### Liquid chromatography (LC)

High-performance liquid chromatography (HPLC) is a commonly used liquid chromatography (LC) technique in quantitative cannabinoid analysis (Hazekamp et al. 2009) (Table 3). The most common columns used in HPLC consist of C18 stationary phases (Citti et al. 2018; Leghissa et al. 2018a) and methanol with 0.1% formic acid or water with 0.1% formic acid as mobile phases (Leghissa et al. 2018a). C18 columns have high resolution and can differentiate between cannabinoids (Citti et al. 2018; Citti et al. 2016). The use of formic acid in the mobile phase provides better peak shape and results than other mobile phases and improved resolution in the chromatographic analysis (Citti et al. 2016). Peschel and Politi (2015) ran two HPLC assays to identify major and minor cannabinoids. Extract profiling was based on the main cannabinoid (THC, CBD, CBG, and CBN) quantification and the presence of acids and flavones. In this research, they found good resolutions of THCA, CBGA, CBDA, THCVA, THC, CBG, CBD, and THCV by HPLC.

**Table 3:** Key properties of common HPLC methods for analysing cannabinoids in eight different studies

Column properties	Mobile phase	Cannabinoids analysed	Flow rate mL/min	Injection volume	Range	LOD	LOQ	References
C18 column	Methanol and water, acidified with formic acid	THCA, CBGA, CBCA, THC, CBG, CBC	0.5	2 μL	200–400 nm	N/A	N/A	(Romano and Hazekamp 2013)
C18 with a C18 guard column	Methanol and water, acidified with formic acid	16 major cannabinoids	0.3	Not stated	280–650 nm	N/A	N/A	(Hazekamp et al. 2005)
C18 with a C18 guard column	Methanol and water, acidified with formic acid	Δ^9^-THC, CBD, CBN, CBG, THCA, CBGA, CBDA	1.5	Not stated	200–400 nm	N/A	N/A	(Hazekamp et al. 2009)
C18 column	0.1% formic acid in both (A) H2O and (B) ACN	CBDA, CBGA, CBD, CBG	0.4	3 μL	2.5–200(μg/mL)	0.5–0.8 (μg/mL)	1.8–2.5(μg/mL)	(Brighenti et al. 2017)
RP18 end-capped column with a guard column	15% of acetic acid in water and methanol	CBG, CBD, CBDA, CBN, CBGA, THC, CBC, THCA	1.5	50 μL	220–280 nm	N/A	N/A	(Namdar et al. 2018)
C18 column	0.1% formic acid in both (A) H_2_O and (B) ACN	CBDA-CBD	0.4	3 μL	> 0.999	0.4–0.1 μg/mL	1.3–0.3 μg/mL	(Pellati et al. 2018)
Silica column, with guard column	Hexane/2-propanol/dimethyl propane (1000/5/1, v/v/v)	N/A	1.0	20 mL	200–440 nm	N/A	N/A	(Oomah et al. 2002)
C18 end capped column with guard column	15% of acetic acid in water and methanol	CBG, CBD, THC, CBC, CBN	1.5	50 μL	220–280 nm	N/A	N/A	(Namdar et al. 2019)

From Table 3, it is clearly obvious that C18 is the most popular column as it is mentioned earlier. The main difference between HPLC and GC is the operating temperature. That is why HPLC is used when preserving the acidic form of cannabinoids are matter. The only disadvantage of HPLC is, it is not able to analyse the volatile compounds like terpenes.

#### HPLC-UV/DAD/MS

Different detection techniques can be used in conjunction with High Performance Liquid Chromatography (HPLC) to analyze cannabinoids. Common detection methods include mass spectrometry (MS) and ultraviolet (UV) absorbance (190 to 400 nm) (Aizpurua-Olaizola et al. 2014; Leghissa et al. 2018a). UV detection is much less expensive and more straightforward than MS detection (Leghissa et al. 2018a). Acidic cannabinoids show absorption peaks at around 270 nm and 310 nm while neutral cannabinoids show absorption peaks at about 220 nm (Citti et al. 2016; Hazekamp et al. 2005). Citti, Ciccarella (Aminah Jatoi et al. 2002) developed a rapid HPLC technique with UV detection (HPLC-UV) to qualify and quantify major cannabinoids (CBDA, CBD, CBN, THC, and THCA) in cannabis extracts. However, absorption profiles from UV detection do not contain enough information to be used in isolation to accurately identify cannabinoids (Leghissa et al. 2018a). Much more information can be obtained by diode array detection (DAD), which covers the visible and UV spectrum. DAD can help to improve specificity because acidic and neutral cannabinoids have different absorption spectrums (Aminah Jatoi et al. 2002; Leghissa et al. 2018a). Thus, Peschel, Politi (Andreae et al. 2015) used HPLC-DAD to differentiate between *Cannabis sativa* chemotypes, extracts of different polarity, and to profile extracts.

Nonetheless, all light absorbance detectors lack the specificity of MS (Citti et al. 2018; Leghissa et al. 2018a), which is particularly useful in analyzing extracts from complex matrices such as cannabis*.* However, some cannabinoids, such as CBG and CBD are difficult to separate using UV detection especially in concentrations greater than 10% in the extract (Citti et al. 2018; De Backer et al. 2009). In the case of CBG and CBD, MS is preferred because it can differentiate between different cannabinoids based on the *m/z* value of their molecular ion (Citti et al. 2018). *M/z* value is not always unique, however; in an ongoing study, Citi, Braghiroli (Beal et al. 1995) found five cannabinoids with the same *m/z* of 315.2294; this value matches that of THC and CBD in Bediol® oil and ethanol extracts. Because some of these cannabinoids coelute, analysis of these compounds is difficult.

#### HPLC-ESI-qTOF/MS

HPLC-electrospray ionization-quadrupole time of flight (HPLC-ESI-qTOF) is very effective in identifying complex and common compounds and can identify the main component of the sample in addition to enhancing the signal to noise ratio in the peaks (Aminah Jatoi et al. 2002). Citti, Ciccarella (Aminah Jatoi et al. 2002) analyzed cannabinoid concentrations in olive oil, ethanol, and supercritical CO_2_ and found that UV-DAD and qTOF detectors produced similar results, thus suggesting that these two detection systems are equally useful in cannabinoid analysis. Pellati and Brighenti (Brighenti et al. 2017; Pellati et al. 2018) used HPLC-ESI-MS both in positive and negative ion mode for the analysis of cannabinoids. By developing HPLC methods, they improved resolution, peak shape, and separation performance together with the improvement of the ionization in HPLC-ESI-MS (Brighenti et al. 2017; Pellati et al. 2018).

#### HPLC-MS/MS

A solution to analyzing co-eluting cannabinoids is to use HPLC-MS/MS (Aizpurua-Olaizola et al. 2014; Citti et al. 2018). Aizpurua-Olaizola, Omar (Borgelt et al. 2013) utilized HPLC-MS/MS to identify THCA, THC, CBD, THCV, CBG, and CBN in 30 cannabis plant varieties. Using the results from their study, they were able to distinguish cannabis plants grown indoors from those grown outdoors. These results suggest that HPLC can be used to successfully determine several cannabinoid profiles and that this method can be used to distinguish between cannabis varieties and growing conditions (Borgelt et al. 2013).

#### LC-MS/MS and APCI

Grauwiler, Scholer (2007) developed a method to simultaneously detect five cannabinoids in human plasma and urine using high-performance liquid chromatography-tandem mass spectrometry (LC-MS/MS) and atmospheric pressure chemical ionization (APCI). Their method had a 25-min runtime with a 0.2 ng/mL lower limit of quantification on samples following human oral administration of 20 mg of THC. Although APCI methods are less sensitive than ESI methods, APCI methods were chosen instead of ESI methods because they produced fewer matrix effects. Limits of detection and limits of quantification were found to be acceptable even with APCI methods (Grauwiler et al. 2007).

#### UPLC-qTOF

Ultra-performance liquid chromatography allows researchers to use a thinner column compared to HPLC, and it can be used for particles less than 2 μm which leads to better separation with higher speed than conventional HPLC. Additionally, Aizpurua-Olaizola, Omar (Borgelt et al. 2013) identified seven unknown minor cannabinoids using UPLC-quadrupole time of flight mass spectrometry (UPLC-qTOF). Jung, Meyer (Brighenti et al. 2017) also implemented qTOF in their study to isolate and identify THCA and 12 of its metabolites in rat urine using LC-MS, LC-MS/MS and LC-qTOF MS. The use of qTOF results in increased accuracy of the detected ions, and when analysing extracts acquired from complex matrices using MS/MS allows for increased cannabinoid specificity (Leghissa et al. 2018a).

Although MS offers many benefits, the use of qTOF mass spectrometers is ideal when trying to differentiate between two compounds with different compositions but the same nominal mass (Citti et al. 2018). qTOF mass spectrometers can provide accurate mass identification with a threshold less than 5 ppm for precursor and product ions; this allows for differentiation between isomers of cannabinoids (Aizpurua-Olaizola et al. 2014; Citti et al. 2018) such as Δ8-tetrahydrocannabinol and Δ9-tetrahydrocannabinol which have the same *m*/*z* because these cannot be differentiated by MS (Citti et al. 2018). Such isomers may have different therapeutic properties and may need to be separated for manufacture, so it is important to adopt an analytical technique that can differentiate between them.

#### Matrix-assisted laser desorption ionization mass spectrometry

Matrix-Assisted Laser Desorption Ionization Mass Spectrometry (*MALDI-MS)* is a new method which has been used in some studies for comparison with usual methods such as LCMS and GCMS in identification of cannabinoids metabolites (Beasley et al. 2016). Recently this method has attracted attention because compared to usual mentioned methods, the sample preparation is simpler, a narrower time frame of drug can be detected, and the sample amount is reduced. Beasley et al. (2016) have used *MALDI-MS* to detect the cannabinoids in a single hair sample. In this study, MALDI instrument was consist of MDS Sciex hybrid quadrupole time-of-flight mass spectrometer with an orthogonal MALDI ion source and a neodymium- doped yttrium aluminum garnet laser.

### LC and GC methods comparison

Liquid chromatography (LC) often employs electrospray ionization (ESI) and atmospheric-pressure chemical ionization (APCI) as ionization sources (Grauwiler et al. 2007). These usually only generate a protonated molecule without diagnostic fragmentation; therefore MS/MS is required to obtain diagnostic information when using LC. Additionally, because cannabinoids have phenolic and carboxylic functional groups that are not ionized effectively using ESI or APCI, GC-MS may offer greater sensitivity that LC-MS (Leghissa et al. 2018a). GC × GC provides greater separation power and analysis speed compared to coupled-column techniques such as liquid chromatography-mass spectrometry (LC-MS) (Dallüge et al. 2003).

However, unlike GC, HPLC allows for differentiation between acidic and neutral cannabinoids (Romano and Hazekamp 2013) because it does not require a derivatization step prior to analysis because high temperatures are not involved in the analysis (Aizpurua-Olaizola et al. 2014; Leghissa et al. 2018a). As a result, cannabinoids do not undergo decarboxylation in an HPLC column, so HPLC provides a more comprehensive chemical report of cannabis samples compared to GC. Table 4 shows LOD and LOQ values for some of the most well-known cannabinoids using HPLC and GC-MS.

**Table 4:** limit of detection and limit of quantification for 5 common cannabinoids using both high performance liquid chromatography and gas chromatography for quantification. (Brighenti et al. 2017; Pellati et al. 2018)

	HPLC		GC-MS	
Interest compound	LOD^b^ (μg/ml)	LOQ^b^ (μg/ml)	LOD^b^ (μg/ml)	LOQ^b^ (μg/ml)
CBD^a^	0.20	0.60	0.17	0.56
THC^a^	0.15	0.47	0.16	0.54
CBG^a^	0.18	0.54	N/A	N/A
CBN^a^	0.10	0.31	0.12	0.39
CBC^a^	0.18	0.53	N/A	N/A

^a^*CBD* cannabidiol, *THC* tetrahydrocannabinol, *CBG* cannabigerol, *CBN* Cannabinol, *CBC* Cannabichromene

^b^ *LOD* limit of detection, *LOQ* limit of quantification

Costs is another parameter for comparison these methods. Set up, maintenance and running costs are often important factors in selecting analytical techniques specially in industry settings. The running cost of LC, GC and HPLC are negligible but for equipment, LC is more expensive than GC and both are much more expensive than HPLC. Coupling of mass spectrometry with LC or GC can further increase the costs.

### TLC (thin layer chromatography)

Hazekamp (2013) used the TLC method both for polar and non-polar systems. They used reversed-phase silica gel plates and normal phase silica gel plates for non-polar and polar systems respectively. For more accurate results they used Fast-Blue B salt (4-benzoylamino-2, 5-diethoxy benzene diazonium chloride hemi salt) which is a suitable coloring agent for visualization of cannabinoids at TLC plates. Fast-Blue B can determine acetylcholinesterase, α- and β-glucosidase activity by changing to different colours which come from reacting of FBB with various compounds, however the colors depend on the concentration of constituents. As a result, they found that TLC is useful in rapid screening of many samples for the existence of cannabinoids, however, its performance is lower compared to other separation methods and the reproducibility of TLC depends on some parameters such as relative humidity (Romano and Hazekamp 2013).

### FTIR Fourier transform infrared spectroscopy (FTIR)

Hazekamp et al. (2005) measured cannabinoids with FTIR. They added KBr to the ethanolic solution of cannabinoids followed by vacuum ethanol evaporation because KBr does not show any absorption spectrum in IR region. Additionally, KBr has a 100% transmission window in the range of wave number at the FTIR spectroscopy. The IR spectra were measured in the range of 500 to 4000 cm^− 1^. Compared to UV spectra, IR spectra presented more absorbance peaks (Hazekamp et al. 2005). Mutje et al. (2007) showed the existence of carbonyl and ester groups by the FTIR peak at 1775 and 1725 cm^− 1^ in composite samples of cannabis extract.

### Nuclear magnetic resonance spectrometry (NMR)

Another alternative to GC and HPLC is NMR (Citti et al. 2018; Hazekamp et al. 2014). NMR is accurate and reproducible and unlike GS and HPLC, NMR is not sensitive to impurities, such a chlorophyll or lipids present in the sample (Hazekamp et al. 2014). Hazekamp, Choi (Casiraghi et al. 2018) developed a method for cannabinoid quantification using ^1^H-NMR that does not require chromatographic purification and has a 5-min final analysis time. In that study, they analysed singlets in the δ 4.0–7.0 range in the ^1^H-NMR spectrum and found that their technique was appropriate for the quantification of CBDA, THCA, CBG, CBGA, and possibly other cannabinoids as well. One of the major advantages of this technique is that reference standards are not required, meaning that this method can quantify cannabinoids that lack pre-existing reference standards and therefore cannot be analysed by other techniques. Although the results from NMR are promising, one major disadvantage to NMR is that high resolution instruments are very expensive (Citti et al. 2018).

## Other parameters to consider when selecting a quantification method

**Fig. 2:**
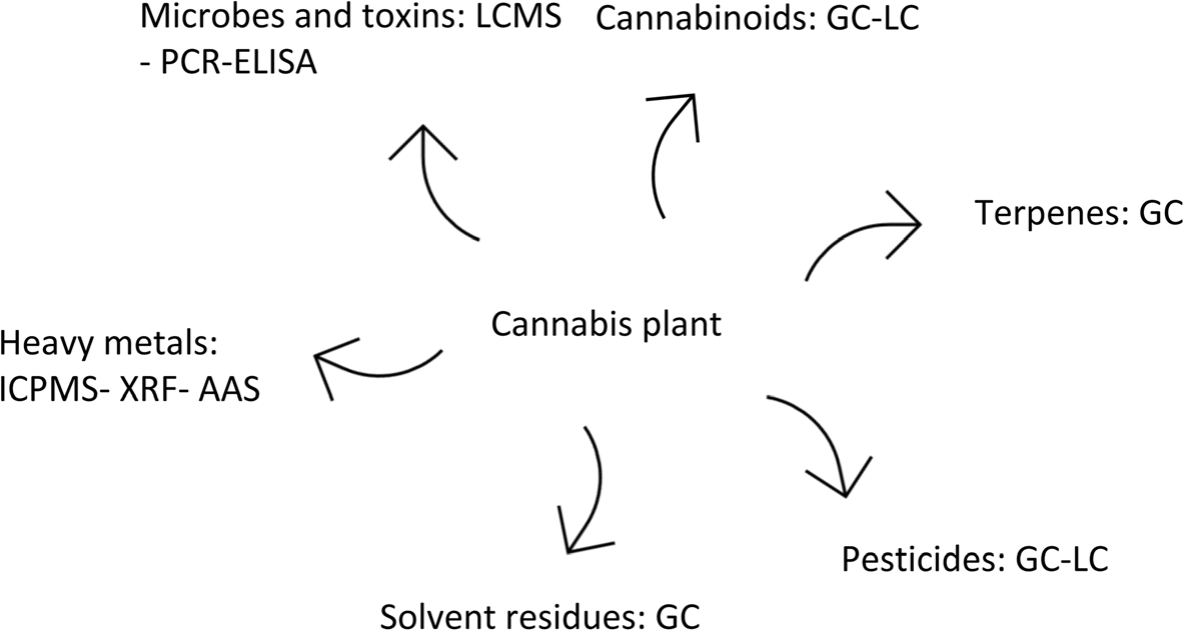
Cannabis compounds and impurities can be detected by various methods. Some methods are suggested in this figure for specific purposes. ICPMS: Inductively coupled plasma mass spectrometry*, XRF: X-ray fluorescence, AAS: Atomic absorption spectroscopy, PCR: Polymerase chain reaction, ELISA: enzyme-linked immunosorbent assay, GC: gas chromatography, LC: Liquid chromatography*

There are other aspects which must be considered when selecting a quantification method such as method performance for different types of cannabinoids and also common interfering substances and impurities. Figure 2 shows suggested methods for cannabis compounds and their impurities. There is no evidence to support which analytical method works best for a specific cannabinoid. However, generally, LC is the preferred method for cannabinoids and GC for terpenes. GC does not have the capability to quantify the acidic form of cannabinoids unless through derivatization while terpenes cannot be detected by LC because they are volatile compounds. Another important factor to consider is analytical sample preparation which is the most time consuming and the most common cause for generating errors during analytical process. Sample preparation method, storage and handling are some of the parameters which can affect the results. For cannabis, the final sample should represent the original lot. So, not only the extraction method, but also the cultivation and processing steps play an important role in the analytical results.

## Conclusion

Given that cannabis preparations from the same cannabis strain can vary by as much as 25% in cannabinoids composition, there is a clear need to develop an effective and efficient cannabinoid quantification technique so that clinicians can be certain about the chemical properties of the products they are administering (Hazekamp and Fischedick 2012). This literature review has explored a variety of cannabinoid quantification techniques.

GC-MS is often employed for cannabinoid quantification (Ciolino et al. 2018; Groger et al. 2008; Hazekamp et al. 2009; Jung et al. 2009; Omar et al. 2013; Omar et al. 2014). However, quantification of cannabinoids via GC requires a derivatization step to avoid the decarboxylation of acidic cannabinoids (Citti et al. 2018; De Backer et al. 2009; Grauwiler et al. 2007; Hazekamp et al. 2009; Leghissa et al. 2018a). Performing GC without derivatization requires the calculation of total cannabinoid content from a combination of acidic and neutral cannabinoid content which can be an uncertain process (Dussy et al. 2005). HPLC-DAD and HPLC-UV provide alternatives to GC analysis but these detection techniques lack specificity and sensitivity (Galal et al. 2009; Grauwiler et al. 2007). The literature suggests that HPLC-MS/MS using ESI and APCI methods provide enough specificity and sensitivity to quantify cannabinoid content in all cannabis extracts (Aizpurua-Olaizola et al. 2014; Citti et al. 2018; Grauwiler et al. 2007).

There are multiple benefits to using HPLC-MS/MS over other analytical methods presented in this review. For example, HPLC can differentiate between acidic and neutral cannabinoids, unlike GC (Romano and Hazekamp 2013). MS offers several benefits over other detection methods as well. For example, MS can differentiate between different cannabinoids based on the *m/z* value of their molecular ion. It offers more specificity compared to UV detectors and can analyze extracts from complex matrices, such as cannabis (Citti et al. 2018; Leghissa et al. 2018a).

## Data Availability

Data sharing is not applicable to this article as no datasets were generated or analysed during the current study.

## Abbreviations

THC: Tetrahydrocannabinol
THCA: Tetrahydrocannabinolic acid
CBD: Cannabidiol
CBDA: Cannabidiol acid
CBG: Cannabigerol
CBGA: Cannabigerolic acid
CBC: Cannabichromene
Δ8-THC: (−)-Δ8-*trans*-tetrahydrocannabinol
CBL: Cannabicyclol
CBND: Cannabinodiol
CBE: Cannabielsoin
CBT: Cannabitriol
CBN: Cannabinol
THCV: Tetrahydrocannabivarin
GC: Gas chromatography
HPLC: High-performance liquid chromatography
LC: Liquid Chromatography
MS: Mass spectrometry
UV: Ultraviolet
FID: Flame ionization detection
DAD: Diode array detection
ESI-qTOF: Electrospray ionization-quadrupole time of flight
APCI: Atmospheric pressure chemical ionization
*UPLC*: Ultra-performance liquid chromatography
TLC: Thin Layer Chromatography
FBB: Fast-Blue B salt
FTIR: FTIR Fourier transform infrared spectroscopy
NMR: Nuclear magnetic resonance spectrometry
GC-VUV: Gas Chromatography with Vacuum Ultraviolet spectroscopy
MALDI-MS: Matrix-Assisted Laser Desorption Ionization Mass Spectrometry
MRM: Multiple Reaction Monitoring
